# NFκB and Kidney Injury

**DOI:** 10.3389/fimmu.2019.00815

**Published:** 2019-04-16

**Authors:** Ning Song, Friedrich Thaiss, Linlin Guo

**Affiliations:** ^1^III. Department of Medicine, University Medical Center Hamburg-Eppendorf, Hamburg, Germany; ^2^Department of Critical Care Medicine, The First Affiliated Hospital of Harbin Medical University, Harbin, China

**Keywords:** NFκB, kidney disease, glomerulonephritis, AKI, autoimmune disease, kidney transplantation, NFκB pathway crosstalk

## Abstract

The global burden of chronic kidney disease will increase during the next century. As NFκB, first described more than 30 years ago, plays a major role in immune and non-immune-mediated diseases and in inflammatory and metabolic disorders, this review article summarizes current knowledge on the role of NFκB in *in vivo* kidney injury and describes the new and so far not completely understood crosstalk between canonical and non-canonical NFκB pathways in T-lymphocyte activation in renal disease.

## Introduction

More than 30 years ago, a protein binding to a specific conserved DNA sequence in nuclear extracts of activated B lymphocyte was identified by Sen and Baltimore (1) and called nuclear factor binding near the κ light-chain gene in B cells (NFκB), although it is not B cell specific, as is now known. NFκB is a protein family consisting of 5 dimers, RelA (p65), RelB, c-Rel, p50 (generated from p105), and p52 (generated from p100), which can form a variety of homodimers or heterodimers. All the subsets share a Rel homology domain that can bind DNA, dimerize and translocate into the nuclear compartment. However, only RelA, RelB and c-Rel have the necessary transcriptional activation domain for target gene expression (2). Normally, NFκB dimers are inactivated through interacting with the inhibitor of κB (IκB) (3). The IκB proteins, including IκBα, IκBβ, and IκBε, preferentially act on different c-Rel and p65 dimers (4), and sometimes, p100 that is not processed to p52 can act as an IκB protein, termed IκBδ (5).

NFκB is regulated by two pathways: the canonical (NEMO-dependent) and the non-canonical (NEMO-independent) pathway. The canonical NFκB pathway always responds rapidly and is mediated by a kinase complex comprising IKKα, IKKβ, and regulatory NEMO (IKKγ). Immune signals, including antigens, Toll-like receptor (TLR) ligand and inflammatory cytokines, such as tumor necrosis factor (TNF) and interleukin 1β (IL-1β), can lead to phosphorylation-dependent activation of this kinase complex. The activated complex will phosphorylate the IκB bound to NFκB dimers (such as p50-p65 and p50-c-Rel), leading to ubiquitination and subsequent proteasome-induced degradation of IκB (6). The degradation releases the NFκB dimers, allowing them to bind κB site-containing DNA and rapidly accumulate in the nuclear compartment. After activation, the synthesized IκB proteins bind to and inhibit NFκB activity and traffic it back to form a negative feedback loop (6).

**Figure 1:**
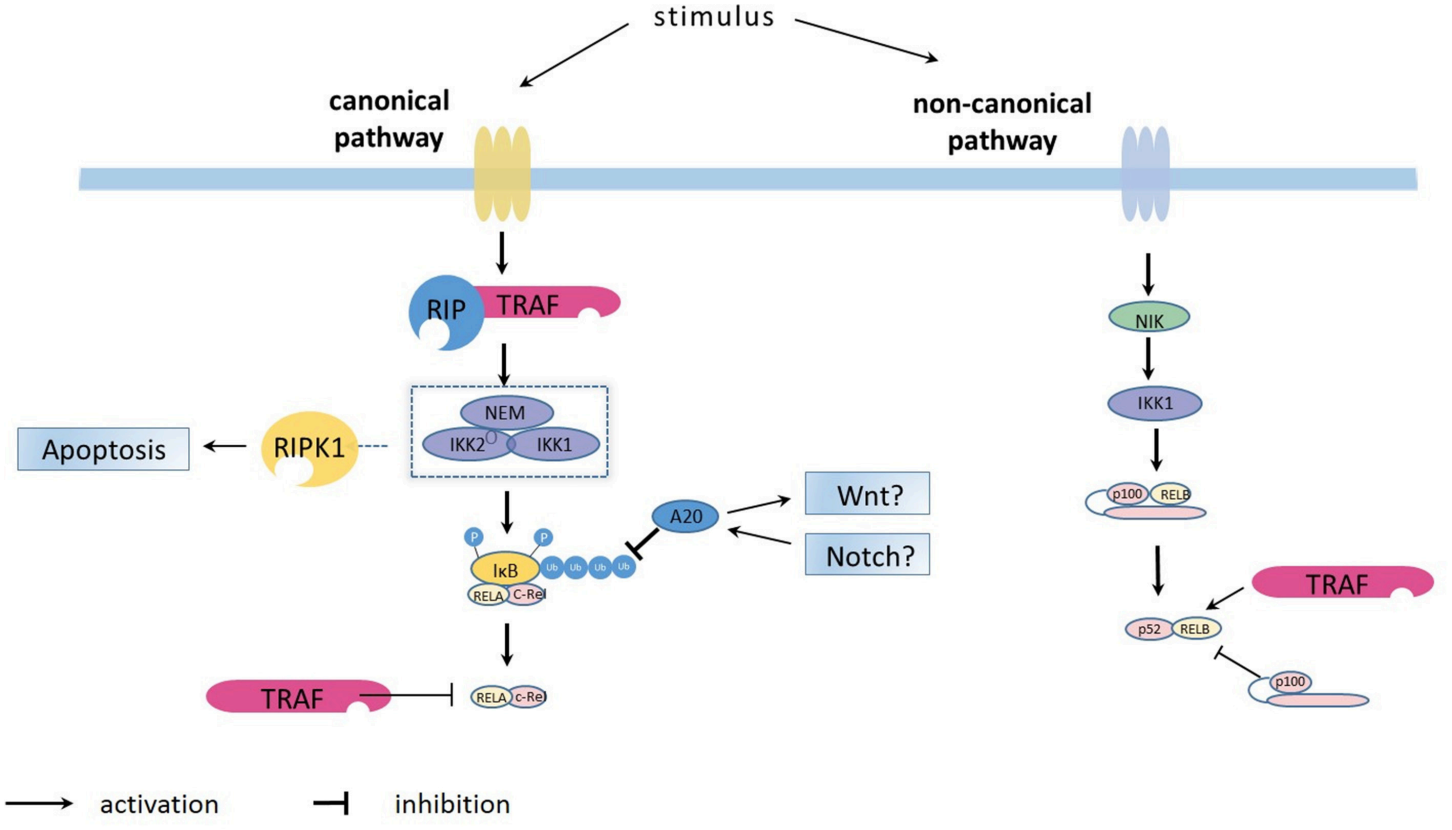
Overview on the principles of canonical and non-canonical NFκB pathways activation. The canonical NFκB pathway is activated by the kinase complex comprising IKKα (IKK1), IKKβ (IKK2) and regulatory NEMO which are for example activated via the TRAF/ RIP complexes (https://doi.org/10.3389/fimmu.2019.00326). The activated IKK1/IKK2/NEMO complex phosphorylates the IκB bound to NFκB dimers (such as p50-p65 and p50-c-Rel), leading to ubiquitination and subsequent proteasome-induced degradation of IκB. The degradation releases the NFκB dimers, allowing them to bind κB site-containing DNA and rapidly accumulate in the nuclear compartment. After activation, the synthesized IκB proteins and also TRAF bind to and inhibit NFκB activity and traffic it back to form a negative feedback loop. The ubiquitin-editing enzyme A20, regulated by the WNT and Notch pathways, represses NFκB function by deubiquitinating of IκB. The non-canonical NFκB pathway is activated through NFκB-inducing kinase (NIK, or MAP3K14), which phosphorylates IKKα (IKK1) which triggers the generation of p50-RelB via proteolytic processing of p100 and binding to RelB.

The non-canonical pathway can be stimulated by members of the TNF cytokine family (such as CD40 ligand, TWEAK and lymphotoxin-β) (7, 8) and membrane attack complexes (MACs) (9). Compared with the constitutive processing of p105 in the canonical pathway, the activation of the non-canonical pathway is based on the processing of p100. This process is accomplished via phosphorylation and subsequent ubiquitination of p100. An important protein, NFκB-inducing kinase (NIK, or MAP3K14), plays a role in phosphorylation through activation of kinase IKKα (10, 11). Once activated, IKKα will trigger the generation of p50-RelB via proteolytic processing of p100 and binding to RelB. The activated NFκB dimers translocate into the nucleus, bind to the DNA and have a major effect on lymphoid organogenesis (Figure 1).

Ubiquitination plays a major role in controlling many transcription factors, such as NFκB. As a reversible and dynamic event, ubiquitination is involved in the upstream activation of both the canonical and non-canonical NFκB pathways (12). However, post-translational modifications, including ubiquitination, are also critical in regulating the NFκB transcriptional response. Stimulus-dependent induction of these modifications can affect the ability of NFκB dimers to bind DNA, interact with IκB proteins, recruit essential co-activators and alter the stability of the proteins. For example, after stimulation of TLRs, the linear ubiquitin assembly complex (LUBAC), which is composed of heme-oxidized IRP2 (HOIL-1L), HOIL-interacting protein (HOIP) and SHANK-associated RH domain-interacting protein (SHARPIN), is recruited by TRAF6 and target NEMO to activate the NFκB pathway (13, 14). However, it is now apparent that ubiquitination and proteasomal degradation of NFκB subunits also represents a major limiting factor in the NFκB transcriptional response (12, 15) (see also below).

Upon activation, NFκB dimers are transported to the nuclear compartment and regulate gene expression by binding to enhancer elements and recruit chromatin remodeling complexes to enhance transcription (16, 17). RelA, for example, recruits acetyltransferases or histone deacetylases after phosphorylation to form the p300/CBP complex (18). c-Rel dimers may remove repressive H3K9 dimethyl modifications from gene promoters to promote pro-inflammatory gene expression. RelA at the same promoter may drive divergent phenotypes by modifying transcriptional noise by targeting chromatin changes (19). NFκB may also synergistically regulate transcription with other transcription factors (12). Recent studies have shown that during activation of key inflammatory genes, NFκB and IRF3 show a gene-specific cooperation in the transcriptional regulation of these genes (20, 21).

In the canonical pathway, IκBs inhibit NFκB dimers and restore the resting state. There are several IκB proteins, including IκBα, IκBβ, and the inhibitory regions on both p100 and p105. As the predominant inhibitory protein, IκB can not only bind NFκB dimers in the cytoplasm, but can also translocate into the nucleus and halt transcription through binding with dimers. On the other hand, dimers that only contain p50 or p52 without transactivation domains repress active dimer binding (22). Upstream of the IκBs, the IRAK-M and several NLRs (NLRP12, NLRX1, and NLRC3) can also negatively regulate NFκB by inhibiting key components in the pathways (23). In the process of posttranscriptional regulation, some micro-RNAs (miRNAs), for example, miR-146a and, miR-302b, and some RNA-binding proteins, such as tristeraprolin (TTP), Regnase-1 and Roquin, regulate the stability or translation of mRNAs encoding specific signaling adaptors of the NFκB pathway (24).

In the negative regulation of NFκB, deubiquitylation plays also a vital role. The ubiquitin-editing enzyme A20 (TNFAIP3) represses NFκB function by deubiquitinating several intermediate NFκB signaling molecules, such as RIP1, TRAF6, and MALT1 (25). Other DUBs, such as CYLD, OTULIN, and cellular zinc finger anti-NFκB (Cezanne) (23), also negatively regulate the NFκB pathway in specific cells. More recently, a novel function for IKKα has been described: it nucleates the ubiquitin-editing enzyme A20 complex, thus enhancing the negative regulatory function in canonical NFκB signaling (26).

The NFκB pathway, especially the canonical one, dynamically regulates the response to external stimulation. In the presence of IκBs and the A20 negative feedback loop, activated NFκB shuttles between the nucleus and the cytoplasm and causes the oscillation of transcription activation and inactivation. For example, different intervals of pulsatile TNFα stimulation particularly effect the transcription of late genes. On the other hand, stimulation with LPS causes only slow and non-oscillatory NFκB activation (27, 28). Playing a key role in this oscillation, newly synthesized IκB is restored in a distant location in the cytoplasm and acts as a pool for resetting NFκB (29). Moreover, the export of IκB mRNA from the nucleus regulates the persistency of the oscillation, and importation of IκB protein effects the frequency of oscillation (30, 31). Body temperature and the circadian clock also regulate the oscillation of NFκB through both IκBα and A20 (32, 33).

In a single cell population, various stimuli and target genes contribute to different dynamics, but the dynamics of NFκB in an identical population of cells are even more complex and differentially regulated considering the susceptibility and inherent stochasticity of the system in each single cell (34–36). Although NFκB plays a vital role in the activation and development of B cells, it is also of fundamental importance in every cell type, such as in innate immune cells and lymphocytes (37), as well as renal (38), and liver (39) parenchymal cells. NFκB has an effect on multiple diseases, for instance, on inflammatory, metabolic, and autoimmune diseases (24, 40) and cancer (41–43). In this review, we focus on the role NFκB may play in *in vivo* kidney diseases.

## Renal Disease—the Perspective of the Global Burden of Chronic Kidney Injury

Kidney diseases can be induced by numerous causes, such as infection, immune and non-immune injury or autoimmune diseases. Most patients who do not repair from their kidney injury will progress to chronic kidney disease (CKD) and finally develop renal failure. In 2002, CDK was the 17th worldwide leading cause of death. The annual mortality rate due to CKD has increased from 11.6 to 15.8 per 100,000 population from 1990 to 2013. It is projected that by 2030, the global burden of kidney diseases will become the 13th leading cause of death worldwide (44). No matter where CKD originates from, inflammation and the immune system play a vital role in the induction of renal injury.

The pathophysiology of kidney diseases is complex, and some of the most recent relevant mechanisms described are briefly mentioned here. The innate immune system includes pathogen-associated molecular patterns (PAMP) and damage-associated molecular patterns (DAMP) induced on innate immune cells, for example, macrophages, neutrophils and dendritic cells. DAMP and PAMP are recognized by pattern-recognition receptors (PRR) expressed on these cells. Toll-like receptors (TLRs) are members of the PRR family and can be found either on the surface or in the intracellular compartment of cells. TLRs can activate the canonical NFκB pathway, which is responsible for the transcription of pro-inflammatory cytokines and chemokines and may result in the progression of kidney disease (45–47). Adaptive immune components, including various T helper (Th) cells, such as Th1, Th2, Th17, and T follicular (Tfh) cells, are derived from naïve CD4 T cells. Th1 and Th17 and the pro-inflammatory cytokines secreted by these cells are involved in many inflammatory renal diseases (48–51). NFκB functions not only in the transcription of pro-inflammation genes in Th1 and Th17 cells but also promotes their differentiation from naïve CD4 T cells through the interaction with innate immune cells (52). In addition to immune cells, some renal cells, for instance mesangial and tubular epithelial cells, can also promote inflammation through the TLRs-NFκB axis (53–55).

The complement system is also involved in the pathogenesis of kidney diseases (56, 57), in kidney transplantation (58, 59) and in T-cell mediated immune injury (60, 61). The classical pathway induced by IgG1, IgG3 and IgM activates C1q, resulting in the activation of C3 through C2 and C4. The mannose-binding lectin (MBL) pathway activate C2 and C4, but not C1, and the alternate pathway activates C3 directly. All three pathways proceed through C3 and C5, causing the production of C5a and C5b. C5a augments inflammation through attracting the inflammation cells, and by contrast, C5b generates the terminal membrane attach complex (C5b-9) (62, 63). The NFκB pathway plays an important role in renal damage mediated by enhanced complement activation (64).

## Renal Disease—Glomerulonephritis

Glomerulonephritis (GN) includes various forms, such as postinfectious GN, lupus nephritis, IgA nephropathy, anti-glomerular basement membrane (GBM) disease and primary and idiopathic membranous nephropathy (iMN), which result from the interaction between renal cells and the immune system with and without immune complex formation. Antigen-Ig antibody complexes (IC) emerge through circulating immune complexes trapped in the glomerulus or from immune deposits with exogenous or endogenous antigens *in situ* (65). For example, in iMN, most patients, nearly 70%, have antibodies directed against the podocyte membrane antigen phospholipase A2 receptor1 (PLA2R) (66, 67). As another important part of the adaptive immune system in glomerulonephritis, T cells, especially diverse T helper (Th) cells, play a vital role in the induction and maintenance of renal diseases. Accumulating evidence indicates that Th17-cells and their characteristic cytokine IL-17 accelerate the induction of nephrotoxic nephritis (NTN), anti-neutrophil cytoplasmic antibody (ANCA)-associated glomerulonephritis and lupus nephritis (48).

The role of NFκB in *in vivo* models of immune glomerular injury is less clear. TANK-binding kinase 1 (TBK1) can suppress the production of IgA in B cells by accelerating the degradation of NIK, which plays an important role in the NFκB non-canonical pathway (68, 69). Our data showed that in crescentic GN, an inhibitor of IKK2, unexpectedly accelerated the disease, which may have resulted from Treg cell impairment (70). In another experimental model of crescentic GN, deficiency in NFκB1 (p50) increased the severity of acute glomerular injury but NFκB1 did not influence the chronic, fibrotic phase (71). In the podocyte, NEMO can activate proinflammatory signaling and aggravate GN (72). In podocyte-specific NEMO knockout mice, glomerular lesion can resolve faster by changing the podocyte cytoskeletal dynamic independently of NFκB (73). In mesangial cells, for example, in lupus nephritis, inhibition of NFκB blocks cell proliferation through the cyclin D1 pathway (74).

Ubiquitin C-terminal hydrolase L1 (UCH-L1) is a deubiquitinating enzyme that influences the inflammatory response by transcription through the ubiquitin-proteasome system (UPS). NFκB increases UCH-L1 expression in podocytes, which plays a role in immune complex-mediated, but not in non-immune complex-mediated, GN (75, 76). UCH-L1 knockout mice have an exacerbation of kidney injury, probably through the decline of proteasomal activity and the increase in oxidative-modified and polyubiquitinated proteins (77). Disruption of ABIN1, another deubiquitinating enzyme, increases the severity of GN through the increased activation of NFκB in podocytes (78). NFκB also induces the expression of periostin and accelerates kidney injury in GN through the activation of integrin-β3 (79).

In patients with immune-mediated glomerular injury, the activation of NFκB has been shown by immunohistochemistry. NFκB is overexpressed in IgA nephropathy, especially in patients with high proteinuria and reduced renal function (80, 81). In patients with VEGF-targeted therapy, increased expression of NFκB (RelA) can repress the content of c-mip in renal thrombotic microangiopathy (TMA) (82). The immunohistochemical examinations in patients with glomerulonephritis demonstrate RelA expression, while other NFκB proteins such as RelB, p52, or p50 have rarely been described. All these morphological examinations in human biopsy only describe the correlation of NFκB expression and clinical outcome parameters in these patients; more evidence about the direct causal relationship of NFκB and disease is still lacking. Single-nucleotide polymorphisms of NFκB, NFκB-inhibitors and NFκB-kinases have also been associated with glomerular kidney diseases (83, 84).

## Renal Disease—Acute Kidney Injury

Acute kidney injury (AKI) is a syndrome defined by a rapid decrease in glomerular filtration, which may be induced by sepsis, ischemia/reperfusion injury (IRI) or nephrotoxic drugs. It is associated with substantial mortality and morbidity in high-income and in low- to middle-income areas (85). Importantly, AKI not only contributes to short-term adverse outcomes, but the survivors may suffer from chronic kidney (CKD) and even end-stage renal disease (ESRD).

As one of the most important components of the pathogenesis, systematic inhibition of NFκB affects the severity of AKI. In a disease model induced by folic acid, inhibition of NFκB mitigates AKI-injury by reduction of RelA and NFκB2 activation (86). In another experimental model of AKI, inhibition of IκB kinase ameliorates injury and improves kidney function even if started 24 h after initiation of AKI (87). NFκB plays its role through immune and epithelial cells in AKI, linking the coordination between inflammation and cell death (88). In innate immune cells, suppression of NFκB in macrophages through blockade of CD38 alleviates LPS-induced AKI (89). In addition, activation of NFκB can increase the infiltration of M1 macrophages in AKI, which may involve the pattern recognition receptor Mincle (90). There is evidence that inhibition of NFκB, specifically in T cells using IκBαΔN-Tg mice to express a transdominant form of IκBα that constitutively represses the activity of multiple NFκB/Rel proteins, protects kidneys from ischemia/reperfusion injury in mice (91). However, by contrast, data from our group showed that in a similar animal model, lymphocyte-specific knockout of IKK2 or NEMO aggravates the damage through the increased infiltration of Th17 cells (92). This discrepancy may result from differences in T cell subpopulations infiltrating the kidneys or from different NFκB subunit activation.

In NFκB tubular epithelial-specific knockout mice, the decrease in NFκB activation protects mice from ischemic AKI through reduced apoptosis and chemokine expression (93). In response to ischemia- and cisplatin-induced AKI, the anti-inflammatory effect of KIM-1 expression is due to the interaction of KIM-1 with p85 and subsequent PI3K-dependent downmodulation of NFκB, suggesting that KIM-1-mediated epithelial cell phagocytosis of apoptotic cells may protect the kidneys after acute injury by downregulating NFκB (94). NFκB, however, may also have anti-inflammatory properties (95). In the IRI mice model, preactivation of NFκB by LPS alleviates subsequent kidney injury, which is accomplished by the HIF-2α-regulated nitric oxide production (96). However, NFκB activation in podocytes is dispensable in the pathogenesis of IRI (97).

As for the function of NFκB in the long-term outcome of AKI, inhibiting IκB kinase showed that AKI induced fibrosis was reduced within 28 days (87). Another group has shown that the disease severity of unilateral ureteric obstruction (UUO) in NFκB1^−/−^ (p50) animals was equivalent with that in wild-type animals (71). Tumor necrosis factor-like weak inducer of apoptosis (TWEAK), which promotes inflammation via NFκB/STAT3 pathways, may also play a major role in the long-term outcome of AKI by activating fibroblast growth factor-inducible-14 (Fn14) receptor, as has been reviewed recently (98). Single-nucleotide polymorphisms of NFκB and NFκB kinases have also been associated with AKI (99).

## Renal Disease—Metabolic Kidney Injury

Obesity is intensely related to type 2 diabetes mellitus (T2D), cardiovascular disease, fatty liver disease and obesity-associated cancers. These diseases are assembled as “metabolic syndrome”. The pathology of all these diseases emerges from insulin resistance and the constantly chronic, low-grade inflammation that is caused by the activation of various inflammatory signals, such as NFκB, JNK, and inflammasomes (100). The kidney is susceptible to diabetes and obesity, with functional impairment including increased proteinuria and glomerular hyperfiltration (101). Therefore, treating chronic, low-grade inflammation by an anti-inflammatory therapy may be a good strategy for T2D-associated kidney injury.

A role of celastrol, an NFκB inhibitor, on insulin resistance and renal function has been demonstrated in diabetic *db*/*db* mice (102). In these experiments, celastrol treatment reduced the creatinine levels, albuminuria, glomerular matrix expansion as well as renal lipids and insulin resistance (102). In another study, peritoneal injection of miR-451 improved renal function in diabetic nephropathy, and the effect was accomplished by the suppression of the LMP7/NFκB pathway-mediated proinflammatory molecule expression (103). Downregulation of NFκB can also ameliorate kidney injury through the reduction of the glomerular NLRP3 inflammasome in diabetic nephropathy (DN), which has been demonstrated in experiments exploring the mechanism of pioglitazone in DN (104). In addition, it was found that in T2D, enhanced NFκB activation impaired vascular function, including myogenic tone, vascular reactivity and inflammatory response, through the detection of PARP-1, SP-1, and COX-2 activity (105). Resolvins, naturally occurring polyunsaturated fatty acids, have anti-inflammatory actions in several tissues, including the kidneys. Resolvins exert this anti-inflammatory effect in diabetes by suppressing inflammatory responses via at least five molecular mechanisms: inhibition of the nucleotide-binding oligomerization domain protein 3 inflammasome, inhibition of nuclear factor κB molecular pathways, improvement of oxidative stress, modulation of nitric oxide synthesis/release and prevention of local and systemic leukocytosis (106). In addition, sirtuin-1 (SIRT1) has a protective role in diabetic kidney disease through the deacetylation of transcription factors such as p53, FOXO, RelA/p65NFκB, STAT3, and PGC1α/PPARγ (107). Taken together, these studies show that NFκB may play a dominant role in the pathogenesis of diabetic nephropathy and metabolic renal diseases (108).

## Renal Disease—Autoimmune Kidney Injury

Autoimmune diseases result from the loss of tolerance to endogenous self-antigens, leading to a varied range of chronic inflammatory conditions that result in substantial morbidity and mortality. Immunosuppressive therapy only improves outcomes in some of the patients with autoimmune diseases and may be accompanied by undesirable treatment side effects. To maintain immune self-tolerance, thymic T-cell selection, Treg cell homeostasis and the elimination of autoreactive B-cell clones are essential, and in all these processes, NFκB subsets play a vital role (109). There is no NFκB inhibitor used for the treatment of autoimmune diseases yet, but various promising approaches targeting diverse stages of NFκB, including IKK, IκB, the ubiquitin-proteasome system, DNA binding, and posttranscriptional and posttranslational modifications are being investigated (109, 110).

A20, A20-binding inhibitor of NFκB (ABIN1), and their genes TNFAIP3 and TNIP1, respectively, are the focus of recent promising therapies. In an experimental model of lupus nephritis (LN), knock-in mice expressing an inactive form of ABIN1 developed progressive GN related to class III and IV LN in humans (83). In addition, ABIN1-deficient mice developed a progressive, lupus-like inflammatory disease characterized by expansion of myeloid cells and leukocyte infiltrations in different parenchymatous organs. These results demonstrated that ABIN1 is an essential anti-inflammatory component of TLR-signaling pathways that controls C/EBPβ and NFκB activity (111).

NFκB may also affect inflammatory autoimmune diseases through the regulation of Tregs. The NFκB family of transcription factors, in particular the canonical pathway subunits, c-Rel and p65, are crucial for the development, maintenance, and function of Tregs. These observations highlight canonical NFκB signaling as a master regulator of Treg development and function, and demonstrate the discrete functions of individual NFκB subunits in Treg-dependent immune tolerance (112–114). The role of the alternative, non-canonical NFκB pathway components, p100 and RelB, in Treg biology was less clear until recently (115). In the latter study, conditional deletion of the p100 gene, nfkb2, in Tregs, resulted in massive inflammation because of impaired suppressive function of nfkb2-deficient Tregs. Surprisingly, deletion of both relb and nfkb2 rescued the inflammatory phenotype, demonstrating an essential role for p100 as an inhibitor of RelB in Tregs (115). In another experiment about the alternative NFκB pathway, NIK SMI1 was shown to be a potent small-molecular inhibitor of NIK, which selectively repressed the non-canonical NFκB pathway and mitigated SLE in mice (116).

Analyses of renal biopsies from patients with different types of lupus nephritis showed increased expression of the transcription factor Nrf2. In an experimental model, Nrf2^−/−^ mice suffered from greater renal damage and more severe pathological alterations in the kidney after the induction of a lupus-like disease. Knockdown of Nrf2 expression aggravated the nuclear factor-kappa B (NFκB) pathway and increased the levels of reactive oxygen species, iNOS, TGFβ1, and fibronectin. These results suggest that Nrf2 improved lupus nephritis by neutralizing reactive oxygen species and by negatively regulating the NFκB and TGFβ1 signaling pathways (117). In an experimental model of ANCA-induced GN, the activation of p50/p65 heterodimers in endothelial cells stimulated by ANCA-primed neutrophils promoted the development of GN (118). In an investigation of SNPs in patients with LN, polymorphisms of TNIP1 were related to LN in patients of European ancestry and in African-Americans (see above) (83). Biallelic loss-of-function alleles accounts for many early-onset and severe immune deficiencies, but some less severe immune disorders with heterozygous mutation are less well-understood. A recent clinical study showed that heterozygous mutation of IKBKB, which encodes IKK2, led to combined T and B lymphocyte deficiency in patients with acquired immune deficiency (119).

## Renal Disease—Kidney Transplantation

Ischemia-reperfusion injury is inevitable in organ transplantation and may cause renal allograft dysfunction. During cold storage and the inflammatory injury following reperfusion, NFκB is activated and plays an important role. In a mouse model of kidney transplantation, knockdown of RelB by local intra-arterial perfusion of the donor kidneys with siRNA relieved IRI in transplanted kidneys by attenuating inflammatory cytokines and cell apoptosis (120). Dexmedetomidine, a sedative drug used in the clinic, reduced kidney injury after transplantation through the inhibition of the TLR4/NFκB pathway in rats (121).

The long-term outcome of renal allografts is reduced by chronic rejection, presenting as allograft vasculopathy (AV). AV is an intriguing process that involves not only adaptive but also innate immunity, such as NK cells, neutrophils and endothelial cells. Deposition of MAC enhances graft immunogenicity instead of causing cell death directly through activation of the non-canonical NFκB pathway (122). Calcineurin inhibitors (CNI) are used for preventing rejection of transplanted kidneys and eventually are one of the major reasons of chronic allograft dysfunction. There is evidence that suppression of NFκB through neutralizing high-mobility group box1 (HMGB1) in mice mitigates chronic cyclosporine nephrotoxicity (123).

In humans, all the components of the NFκB pathway were shown to be expressed in renal allografts with acute rejection and chronic renal allograft injury through qPCR, but there is no clear functional relationship between the expression of NFκB components and allograft rejection or long-term outcome (124). Spontaneous, clinical operational tolerance, characterized as well-functioning allograft without rejection after recession of immunosuppression, has been associated with lower c-Rel expression and a higher proportion of Tregs in allograft biopsies (125). Higher frequencies of mutant genotypes of tag-SNPs of FOXP3 and NFκB1 occurred in patients with end-stage renal disease and with acute rejection episodes (126). These studies indicate a significant relationship between NFκB canonical pathway activation and rejection episodes, but further information on the role of the non-canonical NFκB pathway in human kidney transplantation is needed.

## Renal Disease—NFκB Pathway Crosstalk

NFκB has comprehensive physiological and pathophysiological effects. However, it does not exist as an isolated signaling pathway. There is intensive crosstalk with many other parallel signaling pathways (127–130). This review will only briefly mention the topic of crosstalk between the NFκB pathways and other signaling pathways but focus on what is currently known on the possible interactions between the canonical and non-canonical NFκB pathways in renal disease.

Tumor necrosis factor receptor-associated factors (TRAFs) and receptor-interacting proteins (RIPs), serving as the signals between NFκB receptors and IKK complexes, are critical not only for the IKK activation but also other pathways as the AP-1 pathway (131). IKKα is involved in tumorigenesis in an NFκB-independent way, as has been shown in colorectal cancer (CRC) (132). In CRC cells, mutant BRAF activates the phosphorylation of the proteolytic fragment of IKKα (p45-IKKα) instead of activation of NFκB and contributes to the CRC proliferation (132). In mice, RIPK1 kinase-dependent cell death can be prevented by the IKKα/IKKβ complex, but not by IKKα nor IKKβ alone, during TNF signaling in an NFκB-independent way. RIPK1 in the TNFR1 complex is phosphorylated directly by IKKα/IKKβ instead of promoting cell death by binding to FADD/caspase-8 (133). Subsequently, this anti-cell death function of IKKα/IKKβ was detected in human cells (134). In research about the effect of temperature on NFκB dynamics, a strong down-regulation of AXIN2, a key factor of the Wnt pathway, is observed, which suggests Wnt/NFκB crosstalk (32). There also exists a distinct activation mechanism of the non-canonical pathway in the defense against pathogens. The complement membrane attach complexes (MAC) form an endosome-based signaling complex with the kinases AKT and NIK through phosphorylation and mediate NIK stabilization instead of a TRAF3-dependent pathway (9). Both the Notch pathway and NFκB effect the development of Tregs, and the Notch signaling pathway crosstalks with the NFκB pathway. In the tumor microenvironment, hyperactive Notch3 enhances the canonical NFκB pathway, regulates the developmental aspects and activity of Tregs, and changes the response to tumors (112). The crosstalk between the NFκB and other parallel signaling pathways has not been addressed in renal diseases in detail (135).

**Figure 2:**
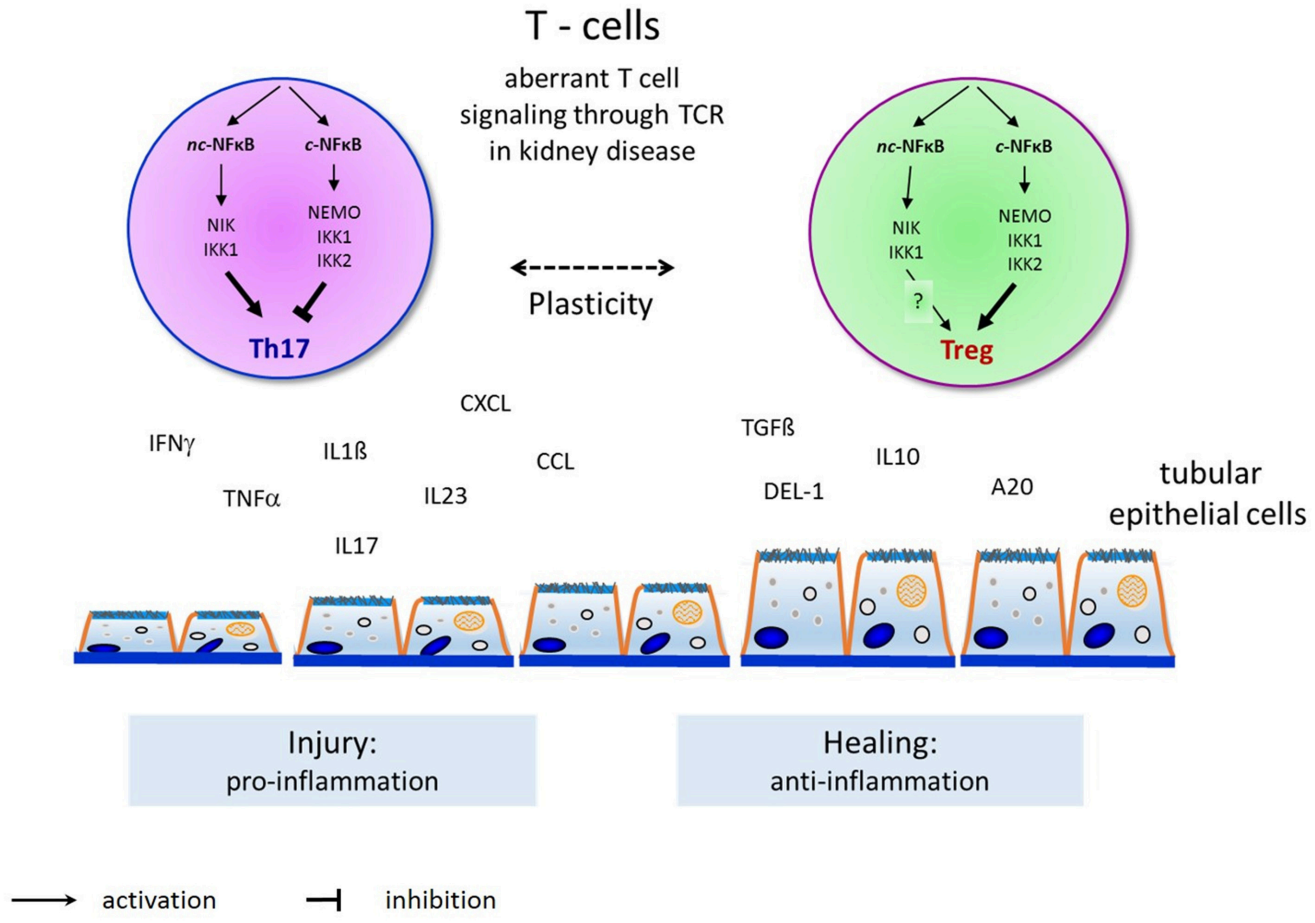
Overview and principles of canonical and non-canonical NFκB pathway activation in T-helper cell differentiation to induce Th17-cells and Tregs and their interaction with endogenous renal cells (such as renal tubular epithelial or glomerular endothelial or mesangial cells) in renal diseases. Th17-cells activation by the non-canonical NFκB pathway induce renal injury (as shown here by tubular epithelial cell damage) through proinflammatory cytokines as shown on the left. Tregs activation by the canonical NFκB pathway permits renal healing (as shown by tubular epithelial cell healing) through anti-inflammatory cytokines on the right.

For now, NFκB has two main activation pathways, the canonical and non-canonical pathways, but the two pathways are not completely separate. A diverse crosstalk occurs at the levels of gene transcription, subunit dimerization, IKK complexes, and upstream signaling and stimulation. For example, the TNF-induced canonical NFκB pathway induces the expression of RelB and inhibits the expression of the CXCL12 gene mediated by the non-canonical pathway (136). Intriguingly, subunits from the two pathways may dimerize and modulate gene expression. For example, in T cells, p52 from the non-canonical pathway cooperates with c-Rel from the canonical pathway to induce the expression of GM-CSF (9). IKKα can activate both the canonical pathway with support from IKKβ and IKKγ and the non-canonical pathway by interacting with NIK. There must exist some unknown mechanisms that activate IKKα in each pathway specifically. Recent results show that the surface structure of IKKα determines the interaction with NIK and the processing of p100 in the non-canonical pathway (137). TNF-like weak inducer of apoptosis (TWEAK) activates both the canonical and non-canonical pathways in different kinds of kidney cells. TWEAK can activate the non-canonical NFκB pathway and promote the expression of CCL21 in podocytes independent of the canonical pathway (138). TRAF2, a common upstream signal in the activation of the canonical and non-canonical pathways, is involved in the protection of cardiac injury through the crosstalk of these pathways by upregulation of RelB as well as diminishing canonical NFκB signaling (139). Crosstalk between the canonical and non-canonical NFκB pathways is essential for medullary thymic epithelial cells to induce central immune tolerance and in Tregs (140). The key function of p100 in Tregs is to be a negative regulator of p52-independent, RelB-containing complexes. These data therefore provide further insight into how constitutive activation of the canonical and alternative NFκB pathways might drive pathology in chronic inflammation and autoimmune disease and demonstrate that the adequate balance of NFκB activity, regulated by NFκB2 p100, is essential for maintaining optimal Treg function and immune tolerance (115). We have recently shown in two experimental models of renal injury that the non-canonical NFκB signaling pathway is critical for Th17 cell induction and that the Th17 cell immune function depends on the fine-tuning of canonical NFκB signaling (92, 141). The crosstalk between canonical and non-canonical NFκB signaling in the complex regulation of Th17 cells and Tregs warrants further investigation to identify potent candidate genes for the manipulation of pathogenicity of Th17 cells without affecting nonpathogenic Th17 and Treg cells that may be critical for tissue homeostasis in renal diseases (109, 142, 143) (Figure 2).

## Conclusion

As a critical regulator of inflammation and cell survival, the NFκB pathway is a promising target for diagnosing and treating kidney diseases. For modulation of the NFκB pathway in the clinic, a number of molecules can effectively inhibit NFκB signaling by targeting the receptors, associated adaptors, IKKs, IκBs and transcriptional regulators (144). There is further clinical evidence on small-molecule inhibitors of IKKα and NIK from recent trials on anti-cancer therapies (145). These clinical trials showed that the cancer-selective pharmacodynamic response of DTP3, the co-inhibitor of the JNK-activating kinase MKK7, and the NFκB-regulated antiapoptotic factor GADD45β could be of clinical value through GADD45B expression in multiple myeloma, which was confirmed to be effective in a subsequent clinical trial (146). However, considering the complexity of the NFκB pathways and the crosstalk between the pathways, there are numerous NFκB-independent side effects that are unrelated to the on-target effects of the inhibitors (147). Therefore, a more specific regulation of NFκB in the targeted disease must be cell-specific.

In addition, as the NFκB system has a broad impact on all kinds of tissues, modulation of NFκB in diseased cells without effecting normal cells must be guaranteed. Therefore, we also need to focus on an appropriate delivery system that will transfer small-molecular inhibitors or genes to specific cells through cell-specific recognition. For example, in macrophages, the acidic lysosomes, the liposomes, the glucan shell microparticles, and the oligopeptide complex have been used in the treatment of metabolic diseases (148). There are also new and emerging therapeutic opportunities with nanoparticles. For example, in hyperglycemia, gold nanoparticles reduce inflammation and apoptosis through tuberin-mTOR/ NFκB pathway inhibition in macrophages (149). In cancer cells, Zn-CuO nanoparticles can induce autophagy and apoptosis through the NFκB pathway (150). Nanoparticles can target a particular cell type as a carrier when packaged with specific receptors (148). More recently, approaches of targeted therapies for the treatment of inflammatory diseases have been highlighted in kidney disease and other diseases, as well through regulation of the NFκB/NLRP3 axis, regulation of the NFκB signaling pathway by miRNAs to cell-specifically suppress NFκB, and activation of the PI3K/Akt/eNOS pathways or by Bcl3 expression (151–162).

Although it has been known for more than 30 years, much remains to be learned about the NFκB pathway, the crosstalk between the canonical and non-canonical NFκB pathways, the interaction of the NFκB pathway with other signal transduction pathways, and the NIK-, IKK-, and NEMO-dependent but NFκB-proteins independent effects in the gene regulation of renal diseases. However, new and exciting NFκB-specific strategies are now being explored for cancer and metabolic diseases and will open new therapeutic opportunities for disease, including cell-specific and phase-specific oscillatory inhibition of NFκB in kidney injury. A complete understanding of these molecular pathways is important, as this may lead to the development of new, effective therapeutic strategies in renal diseases.

## Author Contributions

FT and NS contributed to the conception and design of the study. NS wrote the first draft of the manuscript. All of the authors contributed to manuscript revision and read and approved the submitted version. The first author, NS takes primary responsibility for communication with the journal and editorial office during the submission process, throughout peer review and during publication. The first author is also responsible for ensuring that the submission adheres to all of the journal's requirements, including, but not exclusive to, the details of authorship, study ethics and ethics approval, clinical trial registration documents and conflict of interest declaration. The first and corresponding author will be available post-publication to respond to any queries or critiques.

### Conflict of Interest Statement

The authors declare that the research was conducted in the absence of any commercial or financial relationships that could be construed as a potential conflict of interest.

## Abbreviations

AKI: acute kidney injury
ANCA: Antineutrophil cytoplasmic antibodies
AV: allograft vasculopathy
CKD: chronic kidney disease
CNI: calcineurin inhibitor
CXCL12: C-X-C Motif Chemokine Ligand 12
DAMP: damage-associated molecular patterns
ESRD: end-stage renal disease
GBM: anti-glomerular basement membrane
GN: glomerulonephritis
Imn: idiopathic membranous nephropathy
IRAK: Interleukin-1 receptor-associated kinase
IRI: ischemia/ reperfusion injury
LP: lupus nephritis
MAC: membrane attach complexes
NLRs: NOD-like receptors
NTN: nephrotoxic nephritis
PAMP: pathogen-associated molecular patterns
PRR: pattern-recognition receptors
RIP: receptor-interacting proteins
T2D: type 2 diabetes mellitus
TBK: TANK-binding kinase
Th: T helper
TLRs: Toll-like receptors
TMA: renal thrombotic microangiopathy
TMA: thrombotic microangiopathy
TRAF: Tumor necrosis factor receptor-associated factors
TTP: tristeraprolin
UUO: unilateral ureteric obstruction.
